# Inhibitory effects of icotinib combined with antiangiogenic drugs in human non‐small cell lung cancer xenograft models are better than single target drugs

**DOI:** 10.1111/1759-7714.14261

**Published:** 2021-12-02

**Authors:** Peng Jiang, Yan Zhang, Jiadong Cui, Xiuxiu Wang, Yu Li

**Affiliations:** ^1^ Department of Pulmonary and Critical Care Medicine, Qilu Hospital, Cheeloo College of Medicine Shandong University Jinan China; ^2^ Department of Pulmonary and Critical Care Medicine Weihai Municipal Hospital Weihai China; ^3^ The Fourth People's Hospital of Jinan Jinan China; ^4^ Department of Pulmonary Medicine Dong'e County People's Hospital Liaocheng China

**Keywords:** bevacizumab, icotinib, microvessel density, recombinant human endostatin (rh‐endostatin), vascular endothelial growth factor A (VEGFA)

## Abstract

**Background:**

This study aimed to evaluate the inhibitory effects and potential mechanisms of icotinib combined with antiangiogenic drugs on lung adenocarcinoma in vivo.

**Methods:**

A total of 72 mouse xenograft models established with human lung adenocarcinoma cells (HCC827) were randomly divided into six groups, including control, icotinib (Ic), bevacizumab (Bev), recombinant human endostatin (En), Ic + Bev and Ic + En groups. Mouse weights and tumor volumes were measured regularly. Half of the nude mice in each group were sacrificed after 16 days of drug treatment. The remaining animals were observed for another 16 days without drug supply. Immunohistochemical staining was performed to detect microvessel density in tumor heart, liver, brain specimens from the nude mice and Ki67 expression. Differential expression of vascular endothelial growth factor (VEGFA) in tumor tissue specimens was determined by ELISA and Western blot.

**Results:**

The results showed that the combined drugs inhibited tumor growth more substantially compared with single drugs, without increasing the toxic effects. The antiangiogenesis effect of the combination was better than that of single drug treatment. In addition, both types of targeted drugs and combination medication not only significantly reduced microvessel density in the tumor tissue itself, but also had a certain impact on decreasing microvessel density in the liver. The combination decreased VEGFA and Ki‐67 amounts significantly more than icotinib or endostatin as a monotherapy.

**Conclusions:**

Icotinib combined with bevacizumab or rh‐endostatin has a stronger inhibitory effect on tumor growth than single‐target drug in vivo, with no additional side effects.

## Graphical abstract

We explored the inhibitory effects and its possible mechanisms of ecotinib combined with antiangiogenic drugs in human non‐small cell lung cancer xenograft models. The results confirmed that the combination therapy was more effective and had stronger inhibitory effects on the microvessel density of tumor tissues. After 16 days of drug withdrawal, the effects of antiangiogenic drugs on the body subsided.

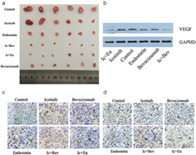

## INTRODUCTION

Lung cancer has become the most common malignant tumor with the highest morbidity and mortality in the world.^1^ About 85% of lung cancer patients are non‐small cell lung cancer (NSCLC) cases, and most of them are lung adenocarcinoma.^2^ Most terminal adenocarcinoma patients harbor epidermal growth factor receptor (EGFR) mutations.^3^ Therefore, epidermal growth factor receptor tyrosine kinase inhibitors (EGFR‐TKIs) have better antitumor effects than traditional platinum‐containing double drug chemotherapy. It has been confirmed by a number of large‐scale clinical trials that first‐generation TKI inhibitors, including gefitinib, erlotinib, and icotinib, significantly prolong progression‐free survival (PFS) in patients with *EGFR*‐sensitive mutations in NSCLC.^4^, ^5^, ^6^, ^7^ Therefore, TKI inhibitors have been recommended by multiple international guidelines as the first‐line treatment for NSCLC patients with *EGFR* mutations. However, these patients often develop progressive disease due to acquired drug resistance within about a year.^8^ Therefore, it is necessary to explore drug combinations to improve the treatment effects and prolong the overall survival of lung cancer patients.

Antiangiogenic drugs including bevacizumab and recombinant human endostatin constitute another group of targeted drugs that block the tumor's oxygen supply and nutritional supplement by inhibiting angiogenesis and indirectly suppress tumor cell proliferation.^9^ They are mainly used in combination with other drugs in clinical trials, since monotherapy with antiangiogenesis targeted drugs has been reported to have only weak therapeutic effects.^10^

Several large‐scale clinical studies have confirmed that the combination of traditional chemotherapy and bevacizumab or recombinant human endostatin can increase PFS and OS in patients with advanced NSCLC,^11^, ^12^, ^13^ In a few clinical trials, bevacizumab combined with EGFR‐TKI erlotinib has also been confirmed to enhance the effect of tumor inhibition.^14^ However, the combination of icotinib with bevacizumab or recombinant human endostatin is rarely reported. Meanwhile, it remains unclear whether the combined application of these two drugs has an interactive effect in addition to their respective antitumor mechanisms. In this study, the antitumor effects of the combination therapy were further evaluated on subcutaneous xenografts in nude mice. In addition, we explored the mechanism of the combined action and the possible interaction. Furthermore, we paid attention to the reaction of the body after the withdrawal of antiangiogenic drugs, about which clinicians are very concerned. It is hoped that this study will provide a reference for clinical decision‐making in patients with advanced lung cancer.

## METHODS

### Cell lines

The human NSCLC HCC827 cell line was obtained from the Cell Bank of the Chinese Academy of Medical Sciences (Shanghai, China). Cells were cultured in RPMI‐1640 (HyClone) supplemented with 10% fetal bovine serum (FBS, Gibco, Thermo Fisher Scientific Inc., NY), 100 U/ml penicillin (HyClone), and 100 μg/ml streptomycin (HyClone) at 37°C in 5% CO_2_.

### Xenograft models and grouping

A total of 72 female BALB/c‐nu athymic mice (5–6 weeks old) were obtained from Nanjing Biomedical Research Institute of Nanjing University. Mice were kept in a standard laboratory under a 12‐h light/dark cycle with ad libitum access to food and water, to acclimatize them for a week before the study. All animal experiments were performed in accordance with the Institutional Guidelines of the Shandong University Animal Care and Use Committee. HCC827 cells (5 × 10^6^ cells/mouse) resuspended in 200 μl of RPMI‐1640 were injected subcutaneously into the right axillary region of the mice. After the tumor had grown to 70–150 mm^3^, the mice were randomized into a control and five treatment groups, including the icotinib, bevacizumab, endostatin, Ic + Bev and Ic + En groups (12 mice in each group). They were treated respectively with vehicle, icotinib (60 mg/kg/day, gavage),^15^ bevacizumab (5 mg/kg/twice a week, i.p.),^16^ recombinant human endostatin (10 mg/kg/day, i.h.),^17^ icotinib plus bevacizumab, and icotinib plus recombinant human endostatin, respectively, for 16 days. Icotinib was provided by Betta Pharmaceutical Inc. Bevacizumab and recombinant human endostatin (rh‐endostatin) were obtained respectively from Roche Pharmaceutical Inc. and Simcere Medgenn Bio‐Pharmaceutical Research Inc.

Bodyweights and tumor volumes of the mice were recorded every 4 days. Tumor volume was derived as V = 3.142(ab^2^)/6,^18^ where a and b are tumor length and width, respectively. After 16 days of drug treatment, half of the nude mice in each group were euthanized, and tissue specimens were collected. Eighteen of the nude mouse tumor tissue specimens and 36 nude mouse heart, liver, lung and brain specimens were fixed with formaldehyde, dehydrated and embedded in paraffin. The remaining half of the specimens were preserved in liquid nitrogen. Drug treatment was discontinued in the remaining 36 nude mice, which were further observed until Day 32. Bodyweights and tumor volumes of the mice were also recorded every 4 days. The remaining nude mice were then sacrificed, and their specimens were collected in the same way.

### Immunohistochemical staining, microvessel density analysis and Ki‐67 staining

Serial tissue sections (4 μm thick) from tumors embedded in paraffin were processed for hematoxylin and eosin (H&E) and immunohistochemical (IHC) staining. Microvessel density (MVD) in tumor tissue and heart, liver and brain specimens of nude mice was evaluated using CD31 immunohistostaining (anti‐CD31 primary antibody, 1:500, Servicebio). Slice areas containing the highest numbers of capillaries and small venules (microvessels, hotspots) were then searched by scanning the tumor sections at low power (40x and 100x). Blood vessels in five different regions of the hot spot were counted under a 200x field of view, and the average value was taken as the MVD.^19^ Any brown‐stained endothelial cell or endothelial‐cell clusters clearly separated from adjacent microvessels, tumor cells and other connective elements were considered as a single countable microvessel. Vessel lumens were not necessary for a structure to be defined as a microvessel. Proliferating cells were assessed by immunohistochemical staining of Ki‐67 (Anti‐Ki‐67 monoclonal antibody, 1:500, Servicebio). The Ki‐67 index (%) was estimated by counting Ki‐67‐positive cell nuclei per 1000 tumor cells in the five regions of the tumor with greatest staining density.

### Enzyme‐linked immunosorbent assay analysis

Tumor tissues stored at −80°C were homogenized with PBS containing complete protease inhibitors. The resulting supernatant after centrifugation at 100 rpm for 10 min was used for the assays. VEGFA levels were determined by an ELISA kit (EBioscience, USA) according to the manufacturer's guidelines. Absorbance was finally read at a wavelength of 450 nm, and VEGFA concentrations were calculated using a standard curve.

### Western blot analysis

Tumor specimens were homogenized and centrifuged at 12 000 rpm for 15 min to collect the supernatant. Then, protein concentrations were measured using a BCA protein assay kit (Servicebio) according to the manufacturer's instructions. Approximately 50 μg of protein was separated by 10% sodium dodecyl sulfate–polyacrylamide gel electrophoresis (SDS‐PAGE) and transferred onto polyvinylidene difluoride membranes (Millipore). Membranes were blocked for 1 h with 5% skimmed milk in Tris buffered saline Tween and incubated overnight at 4°C with primary antibodies directed against VEGFA (1:500, Proteintech Group Inc.). The membranes were then incubated with a horseradish peroxidase (HRP)‐conjugated secondary antibody (1:3000, Servicebio) for 30 min at room temperature. After incubation, the membranes were washed three times for 5 min with Tris buffered saline/Tween, and proteins were visualized by chemiluminescence. Finally, protein signals were quantified by densitometry and analyzed with the ImageJ software (National Institutes of Health).

### Statistical analysis

Quantitative data are expressed as the mean ± standard deviation. The *t*‐test and one‐way analysis of variance (ANOVA) were used for intergroup comparisons. Differences were considered statistically significant for *p* < 0.05. Statistical analyses were performed with SPSS version 22.0 (IBM Corp.).

## RESULTS

### Combination of drugs inhibits the growth of tumor significantly better than single drugs

**FIGURE 1:**
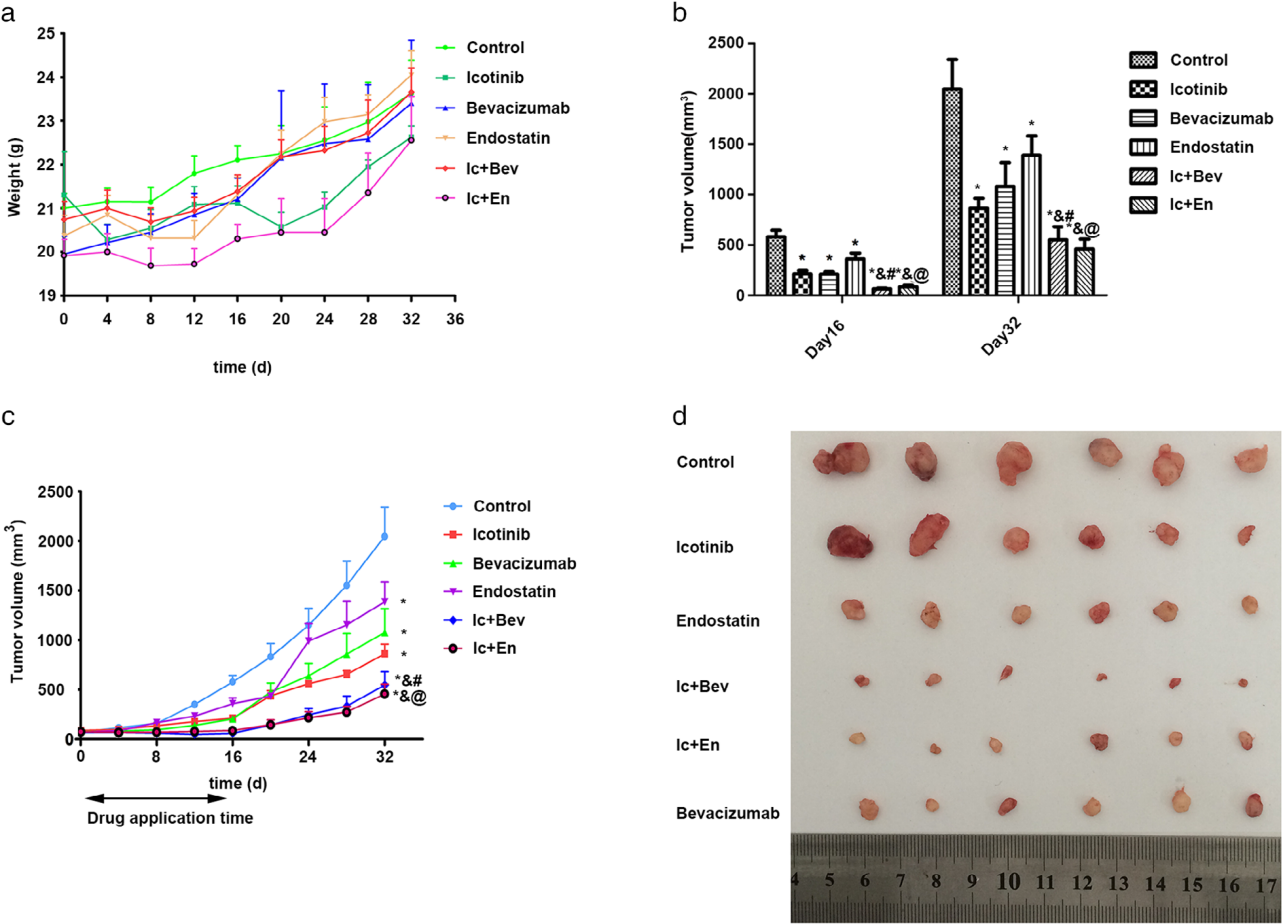
(a) Bodyweight curves in six groups of nude mice during the experiment withdrawal. (b, c) Tumor growth curves in the six groups during drug administration and after drug withdrawal. Data are mean ± SD. (d) Tumor sizes during different groups at the end of the 16th day. Data are mean ± SD. *p < 0.05 versus the control group, &*p* < 0.05 versus the icotinib group. #*p* < 0.05 versus the bevacizumab group, @*p* < 0.05 versus the rh‐endostatin group. Bev, bevacizumab; En, endostatin; Ic, icotinib

Throughout the course of treatment, the diet and daily activities of the nude mice were normal, and there was no significant weight loss (Figure 1). During and after the whole experiment, all the nude mice survived. It was confirmed that the drug combinations did not significantly increase the toxic and side effects. The tumors increased in volume rapidly in the control group, while tumor growth was significantly decreased in all drug treatment groups (*p* < 0.05, Table S1, Figure 1). Further analysis indicated that tumor volumes in icotinib plus antiangiogenesis (Ic + Bev or Ic + En) group were significantly smaller compared with other groups (*p* < 0.05, Table S1, Figure 1). These data demonstrated that the drugs administered to mice inhibited the growth of xenograft tumors, and the combination of drugs showed significantly higher efficacy in tumor growth inhibition compared with other treatment groups.

After drug cessation from Day 16, tumors in the drug treatment group grew more rapidly than previously. After 8 days of drug discontinuation, tumor growth was the fastest in the endostatin group, followed by the icotinib group and bevacizumab group, and the growth was the lowest in the icotinib plus antiangiogenesis (Ic + Bev and Ic + En) groups. Thereafter, the tumor growth rate in each group was consistent until the end of the study (Figure 1). In conclusion, the combined drugs inhibited tumor growth more than single drugs.

### Antiangiogenic effects of drug combinations are stronger than those of single antiangiogenic drugs

**FIGURE 2:**
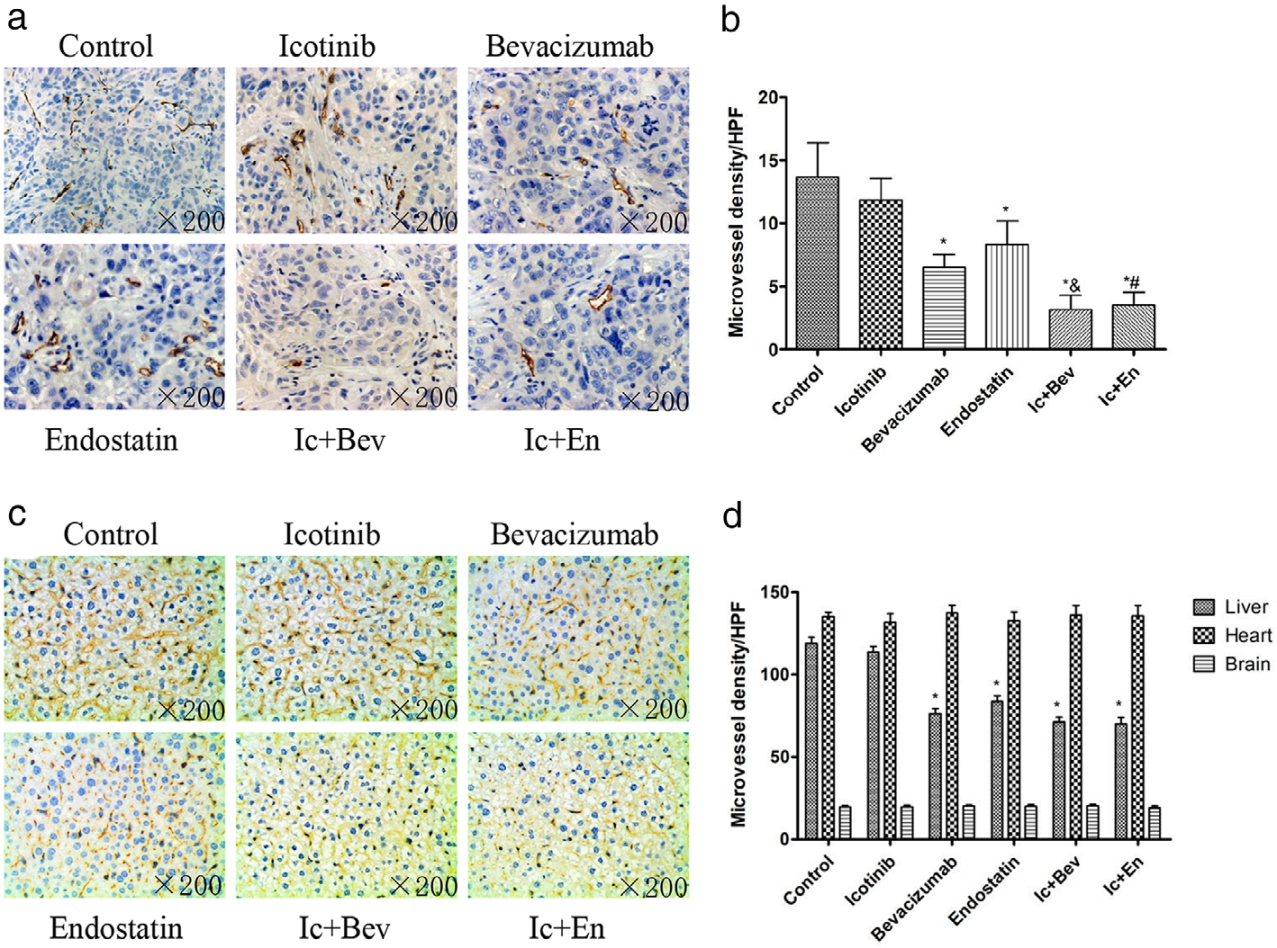
The differential expression of CD31 in the tumor tissue in different groups and different organs at the end of treatment. (a, b) The differential expression of CD31 in tumor tissue of xenograft mice in different groups at the end of treatment. Data are expressed as the mean ± SD. (c) Differential expression of CD31 in hepatic tissues of different groups at the end of treatment. (d) Quantitative analysis of CD31 expression in xenografted mice tissue of liver，heart and brain at the end of treatment. Data are mean ± SD *p < 0.05 versus the control group, &*p* < 0.05 versus the bevacizumab group, #*p* < 0.05 versus the endostatin group. Original magnification, ×200. Bev, bevacizumab; En, endostatin; Ic, icotinib

To explore the mechanism by which antiangiogenic drugs inhibit tumor growth, microvessel density of tumor tissues were measured by IHC. The results showed that microvessel density in treatment groups containing antiangiogenic drugs were significantly decreased compared with those of the control and icotinib groups (*p* < 0.05, Figure 2a,b), although there was no statistically significant difference between the icotinib and control group. In addition, microvessel densities were significantly decreased in the combined drug groups (Ic + Bev and Ic + En groups) compared with the single antiangiogenic drug (bevacizumab or endostatin) groups (*p* < 0.05, Figure 2a,b). In conclusion, the antiangiogenic effects of the combined drugs were stronger than those of single antiangiogenic drugs.

Microvessel densities in different organs, including the liver, heart and brain, were also measured by IHC. The results showed that hepatic microvessel density in drug‐treated groups were significantly decreased compared with control values (*p* < 0.05, Figure 2c,d), except for the icotinib group, as there was no statistically significant difference in hepatic microvessel density between the icotinib and control groups.

However, in the heart and brain, there were no significant differences between drug treatment groups and control animals (*p* > 0.05, Figure 2d).

**FIGURE 3:**
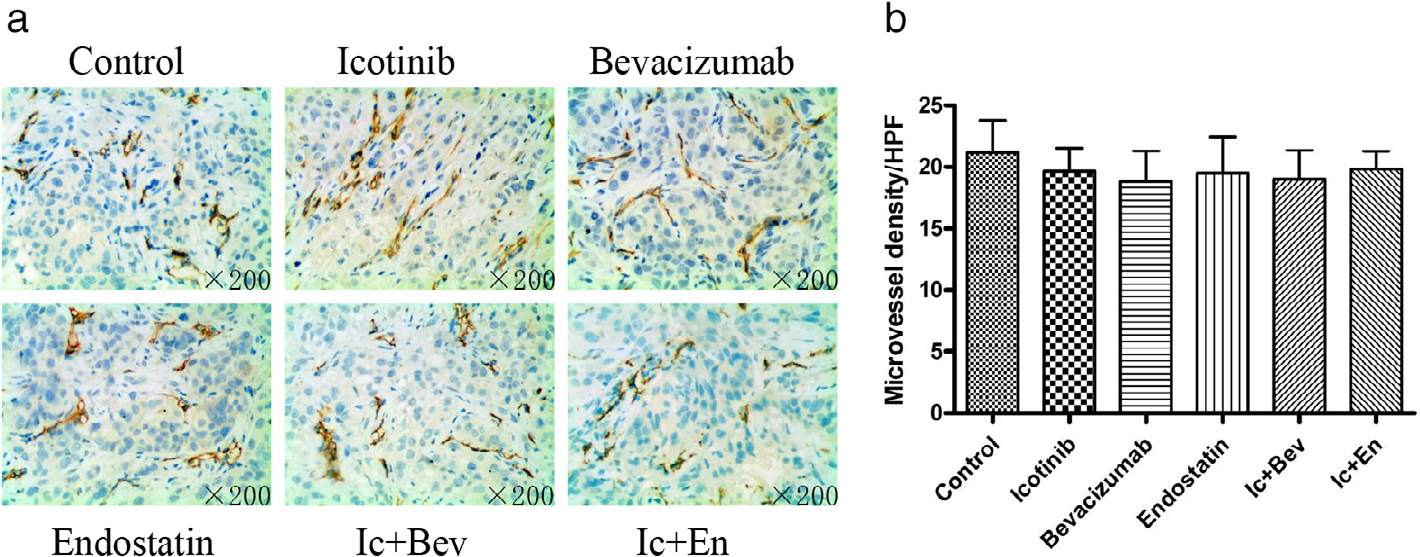
The differential expression of CD31 in the tumor tissue in different groups 16 days after drug withdrawal. (a, b) The differential expression of CD31 in tumor tissue of xenograft mice in different groups at the end of treatment. Data are expressed as the mean ± SD. Original magnification, ×200. Bev, bevacizumab; En, endostatin; Ic, icotinib

Next, we also observed the changes in microvessel density of tumor tissues and other major organs in nude mice 16 days after drug withdrawal. There was no statistical difference in tumor microvascular density between all drug treatment groups and the control group (*p* > 0.05, Figure 3). There was also no statistical difference in microvascular density of the liver, brain and heart organs (*p* > 0.05). This reminds us that, with the withdrawal of the drug, the microvessel density of tumor tissues and various organs in initial drug treatment groups gradually became consistent with the control group. Eventually, the effect of antiangiogenic drugs on the body subsided.

Above all, antiangiogenic drugs not only had a significant impact on the microvessel density of the tumor tissue itself, but also had a certain effect on microvessel density in the liver. However, it had little effect on microvessel density in the heart or brain.

### Combined drugs inhibit the expression of Ki‐67 in the tumor tissue more than single targeted drugs

**FIGURE 4:**
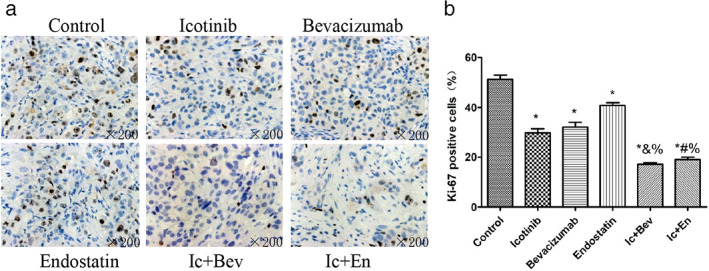
Representative immunohistochemistry images for Ki‐67 detection in different groups at the end of treatment. Data are mean ± SD. *p < 0.05 versus the control group, &*p* < 0.05 versus the bevacizumab group. #*p* < 0.05 versus the endostatin group, %*p* < 0.05 versus the icotinib group. Original magnification, ×200. Bev, bevacizumab; En, endostatin; Ic, icotinib

**FIGURE 5:**
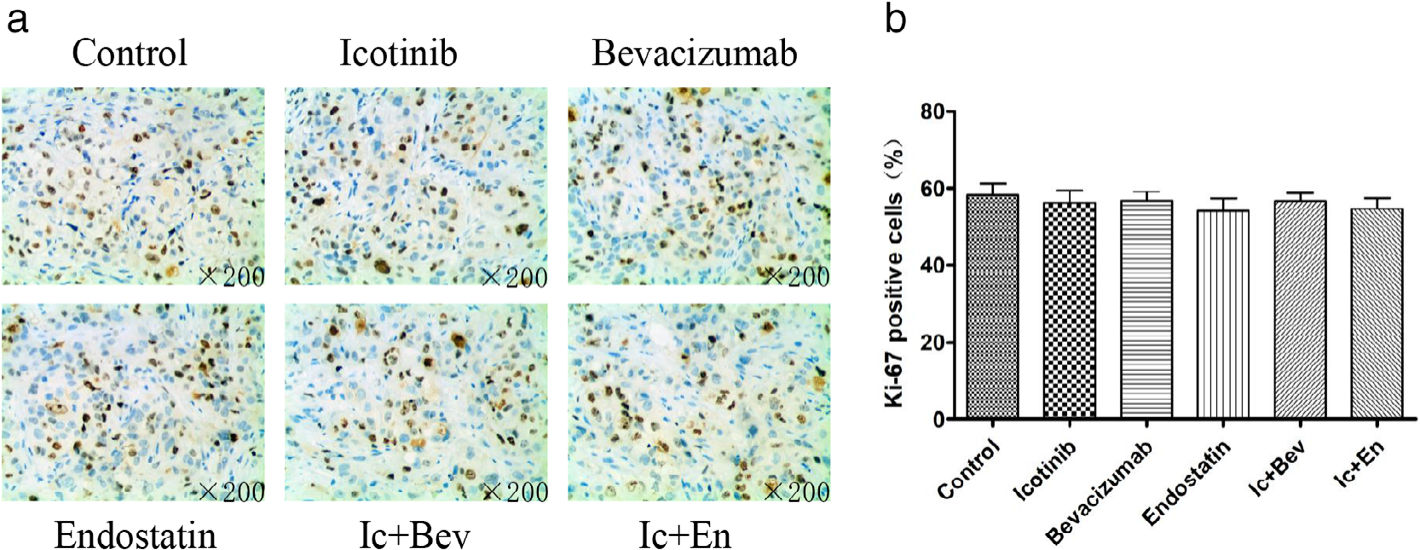
Representative immunohistochemistry images for Ki‐67 detection in different groups 16 days after drug withdrawal. Data are mean ± SD. Original magnification, ×200. Bev, bevacizumab; En, endostatin; Ic, icotinib

To investigate the effects of drugs on cell proliferation, IHC was performed to measure the proportion of Ki‐67‐positive cells. The results showed that the expression levels of Ki‐67 in drug‐treatment groups were significantly decreased compared with control values, and the drug combination groups (Ic + Bev and Ic + En groups) showed significantly decreased values compared with the single drug groups (icotinib, bevacizumab and endostatin groups) (*p* < 0.05, Figure 4). In the subsequent drug withdrawal experiment, we also found that the expression levels of Ki67 in tumor tissues was not statistically significant between the control group and all drug treatment groups (*p* > 0.05, Figure 5). In conclusion, drug combination inhibited tumor cell proliferation better than single targeted drugs.

### Drug combinations decrease the expression of VEGFA compared with bevacizumab and endostatin monotherapies

**FIGURE 6:**
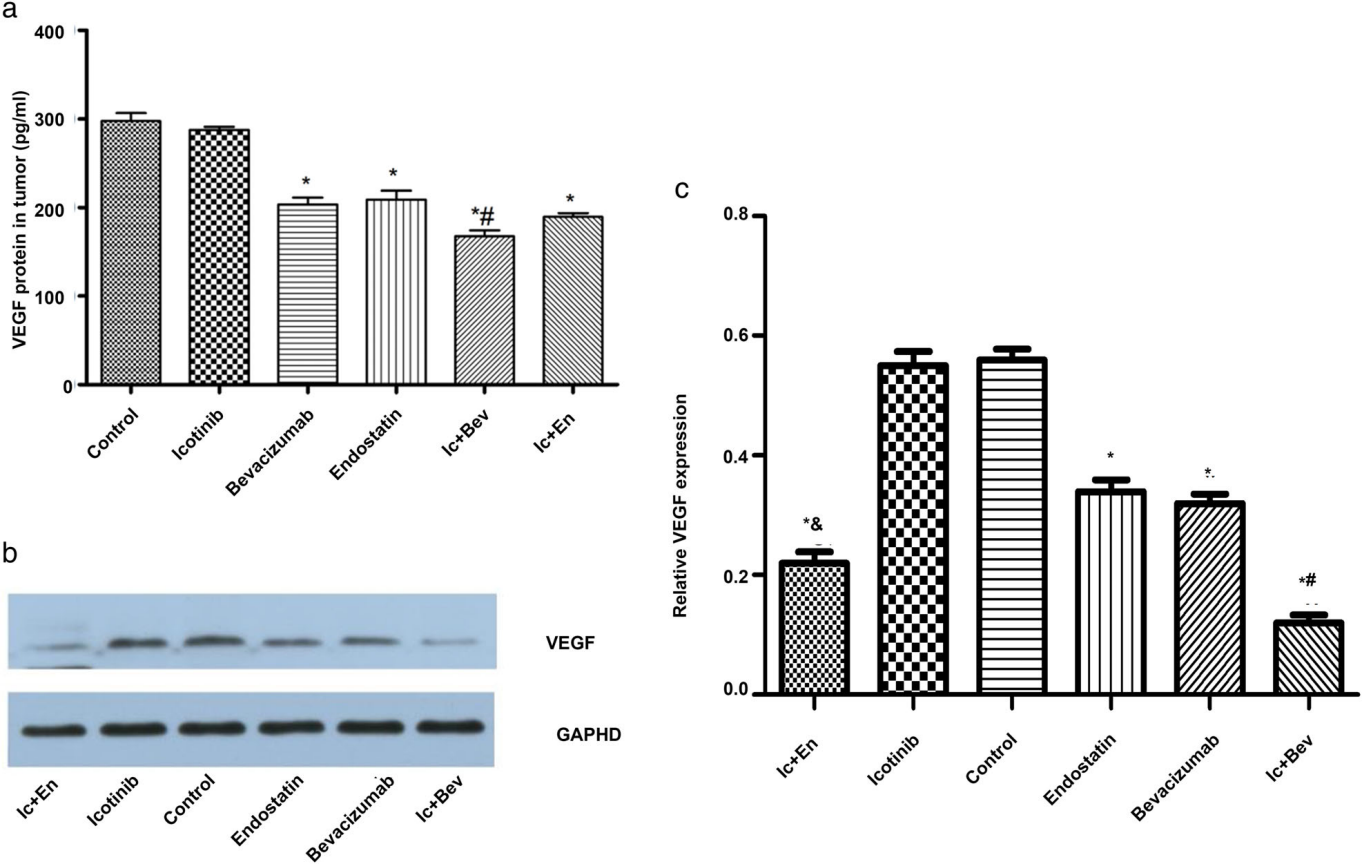
Expression of VEGF determined by ELISA and Western blot. (a) The VEGF concentration in the supernatant of tumor tissues. (b,c) Expression of VEGF (WB) and relative VEGF expression. Data are expressed as the mean ± SD. *p < 0.05 versus the control group. #*p* < 0.05 versus the bevacizumab group. &*p* < 0.05 versus the endostatin group

To further explore the effects of drugs on tumor growth, the amounts of VEGFA were measured by ELISA and WB. The results showed that VEGFA concentrations in tumor tissue supernatants in the drug treatment groups were significantly reduced compared with control values (*p* < 0.05, Figure 6), except for the icotinib group. In addition, VEGFA levels in the drug combination group (Ic + Bev group) was lower than that of the bevacizumab monotherapy group (*p* < 0.05). VEGFA levels in the drug combination group (Ic + En group) were also lower than that of the endostatin monotherapy group, although the difference was not statistically significant (*p* > 0.05, Figure 6).

Consistent with ELISA datas, the expression levels of VEGFA determined by Western blot were decreased in the drug treatment groups compared with the control group. Similarly, VEGFA amounts in the drug combination groups (Ic + Bev and Ic + En groups) were lower than those of the bevacizumab or endostatin group (*p* < 0.05, Figure 6). Taken together, VEGFA was downregulated by antiangiogenic drugs, and the inhibitory effects of combined drugs were better than those of single antiangiogenic drugs.

## DISCUSSION

Molecular targeted drugs have gradually entered the clinical setting for first‐, second‐line and maintenance treatments of lung cancer. It has been reported that bevacizumab^20^ and recombinant human endostatin (rh‐endostatin)^21^ inhibit tumor growth by blocking angiogenesis mediated by vascular endothelium. These two targeted drugs are currently used in combination with third generation chemotherapeutic drugs, but the assessment of joint application of these two drugs with EGFR‐TKI is rare. In this study, we examined the impact of icotinib combined with bevacizumab or rh‐endostatin on subcutaneous xenografts in nude mice. The results showed that combined drugs inhibited tumor growth more efficiently than single drugs, without increasing toxic and side effects. The antiangiogenic effects of the drug combinations were better than those of single drug treatments.

Newborn capillaries play a very important role in the process of tumor growth and metastasis.^22^ Tumor angiogenesis is a very complex system regulated by many factors, among which vascular endothelial growth factor had the most important function. Bevacizumab is a recombinant human monoclonal IgG that can specifically bind to VEGF and inhibit tumor growth. Endostatin, a noncollagen region fragment of XVIIIc and an autocrine angiogenesis inhibitor,^23^ inhibits vascular endothelial growth and exerts antitumor effects. Our results showed that tumor volume after treatment with bevacizumab or recombinant human endostatin alone was significantly reduced compared with the control value. In addition, the combination group (whether combined with bevacizumab or recombinant human endostatin) had better tumor inhibition than the single‐targeted drug and control groups. Meanwhile, drug withdrawal assays also suggested a lower growth rate of tumors in the combination group. A previous study in 2014 reported that the erlotinib plus bevacizumab combination has lower median progression‐free survival compared with erlotinib alone (16 months vs. 9.7 months),^14^ which is consistent with our conclusion. A meta‐analysis in 2016 also showed that EGFR‐TKI combined with antivascular targeting drugs has higher progression‐free survival (PFS) than the targeted drug alone.^24^ Further immunohistochemical analysis of transplanted tumors in nude mice also suggested that the proliferation index Ki67 in the drug combination group was significantly lower than that of the single‐targeted group, leading to slower rate of tumor growth. Targeted drugs combined with antiangiogenic agents may be an effective new option for lung cancer patients with *EGFR* mutations.

One of the mechanisms of vascular targeted drugs combined with TKI therapy is that vascular targeting drugs inhibit blood vessel growth in tumor tissues to reduce blood supply and cause tumor necrosis.^16^ Our experimental results showed that after drug withdrawal, microvascular densities in tumor tissues in the bevacizumab, recombinant human endostatin and combined drug groups, containing antiangiogenic drugs, were lower than those of control animals and the EGFR‐TKI group alone. It confirms that potentiation of EGFR‐TKI by vascular‐targeted drugs exerts effects by inhibiting the growth of tumor blood vessels.^16^

The contents of VEGFA in tumor tissues were further detected by ELISA and Western blot. VEGFA amounts in groups containing antiangiogenic drugs were lower compared with those of control samples and the icotinib group alone. It is well explained in theory because VEGFA is the target of vascular‐targeted drugs.^20^, ^21^ This leads to decreased angiogenesis, which inhibits the growth of tumors. We also found that VEGFA levels were lower in the combination treatment group compared with the antiangiogenic drug group alone. This reduction was more significant in the icotinib plus bevacizumab group compared with the bevacizumab group (*p* < 0.05). There was also a downward trend in groups containing rh‐endostatin. Previous studies have found that gefitinib and erlotinib can downregulate VEGFA.^25^, ^26^ EGFR regulates VEGFA expression via the MAPK and PI3K signaling cascades and at least three different signaling factors, including STAT3, Sp1 and hypoxia‐inducible factors (HIFs).^27^ Therefore, it was speculated that the combination of icotinib and antiangiogenic drugs can lead to further downregulation of VEGFA compared with antiangiogenic drugs alone, with stronger inhibitory effects on angiogenesis. That may be why the combination group had better antitumor effects. Compared with the rh‐endostatin group alone, VEGFA level difference in the icotinib plus rh‐endostatin group did not reach statistical significance (*p* > 0.05). It may be due to the drug dose factor. Another possible explanation is that rh‐endostatin is a broad‐spectrum antiangiogenic drug rather than a specific anti‐VEGFA drug. In addition, previous studies have shown that bevacizumab can change tumor vessel physiology and improve drug delivery,^28^, ^29^ which explains why the combined drug treatment group had stronger antitumor effects. Due to complex biological factors and signaling pathways in the formation of tumors, the improved antitumor effects of the drug combinations are not only because of their respective tyrosine kinase inhibitors and antiangiogenic effects, but also their potential effects and interactions with each other. Further mechanisms of drug combinations need to be explored.

We also observed the changes of tumor volume after drug withdrawal. After 4 days of cessation of antitumor drugs, tumor growth was accelerated in each treatment group. Among them, the combined drug treatment groups had the slowest tumor growth rate, followed by the bevacizumab and the icotinib groups. The rh‐endostatin group had the fastest growth rate. However, the differences disappeared 8 days after drug withdrawal, that is, tumor growth rates in nude mice were similar 8 days after drug withdrawal among all groups. This was also verified by the fact that there were no significant differences in microvascular density and the expression of Ki67 between the drug‐treated and control groups at the end of the observation period. Previous studies have suggested that the sudden withdrawal of antivascular drugs may lead to accelerated tumor growth or increased invasiveness, the so‐called “rebound” phenomenon.^30^ Yet some studies do not support this phenomenon.^31^ Our data suggests that tumor growth in the rh‐endostatin group appears to have a certain degree of “rebound” after drug withdrawal, but this phenomenon was not observed in the bevacizumab group. Further experimental studies are warranted to confirm whether this phenomenon is due to rh‐endostatin as a broad‐spectrum antiangiogenic drug rather than a specific anti‐VEGFA drug, or to other reasons. In summary, the “rebound” phenomenon may occur at the initial stage of discontinuation of antiangiogenic drugs, but such a “rebound” will gradually disappear.

The side effects of vascular antiangiogenic drugs, especially their effects on blood vessels in normal tissues, have always been a concern among clinicians. Our study showed that vascular‐targeted drugs had significant effects on hepatic microvascular density, which quickly recovered to a similar level to that of the control group after drug withdrawal. There was no significant effect on microvascular density in the heart and brain. These results are consistent with Yang et al.^32^ We also observed that all nude mice in the combination groups had good appetite and fitness throughout the course of treatment, and bodyweights were not significantly reduced. There were no deaths of nude mice during the entire treatment and drug withdrawal observation periods. The above findings demonstrate that icotinib plus antiangiogenic drugs has fewer side effects and good safety.

In conclusion, this study examined the effect of icotinib plus antiangiogenic drugs in human NSCLC xenograft models, suggesting that combination therapy is more effective, and side effects are not significantly increased. This drug regimen deserves clinical application.

## CONFLICT OF INTEREST

The authors declare that they have no competing interests.

## Supporting information


**Table S1**. Tumor volumes observed at the end of drug administration and drug withdrawal.
