# Supplementary material for: Inhibitory effects of icotinib combined with antiangiogenic drugs in human non‐small cell lung cancer xenograft models are better than single target drugs
Source: Thorac Cancer. 2021 Dec 2;13(2):257–64. doi: 10.1111/1759-7714.14261 (PMC8758432; doi:10.1111/1759-7714.14261)
Supplement: Supplementary file 1 — Table S1. Tumor volumes observed at the end of drug administration and drug withdrawal. [file TCA-13-257-s001.docx]

**Supplement Table 1.** Tumor volumes observed at the end of drug administration and drug withdrawal

| Group | Tumor volumn(mm^3^, $\bar{\text{x}}\pm\text{s}$) | |
| --- | --- | --- |
|  | d16 | d32 |
| Control | 577.56±65.99 | 2044.77±296.01 |
| Icotinib | 211.90±33.78* | 862.48±96.31* |
| Bevacizumab | 204.37±28.52* | 1077.61±237.63* |
| Endostatin | 359.21±58.77* | 1387.80±194.97* |
| Ic+Bev | 60.80±7.72*^&#^ | 546.38±134.73*^&#^ |
| Ic+En | 85.05±15.01*^&@^ | 456.94±100.32*^&@^ |

**Notes:** Data are expressed as the mean ± SD. *P < 0.05 versus the control group, &P < 0.05 versus the icotinib group. #P < 0.05 versus the bevacizumab group, @P < 0.05 versus the rh-endostatin group.

**Abbreviations:** Ic, Icotinib; Bev, Bevacizumab; En, Endostatin;
